# In Vitro and In Vivo Experimental Studies of PM_2.5_ on Disease Progression

**DOI:** 10.3390/ijerph15071380

**Published:** 2018-07-01

**Authors:** Ching-Chang Cho, Wen-Yeh Hsieh, Chin-Hung Tsai, Cheng-Yi Chen, Hui-Fang Chang, Chih-Sheng Lin

**Affiliations:** 1 Department of Biological Science and Technology, National Chiao Tung University, 75 Boai Street, Hsinchu 300, Taiwan; ccjwo21@gmail.com (C.-C.C.); mict6009@gmail.com (C.-H.T.); ricechen@hotmail.com (C.-Y.C.); olulu789.bt05g@g2.nctu.edu.tw (H.-F.C.); 2 Division of Chest Medicine, Department of Internal Medicine, Hsinchu Mackay Memorial Hospital, 690 Section 2, Guangfu Road, Hsinchu 300, Taiwan; 4040@mmh.org.tw; 3 Division of Pulmonary Medicine, Department of Internal Medicine, Tungs’ Taichung Metro Harbor Hospital, 699 Section 8, Taiwan Blvd., Taichung 435, Taiwan; 4 Division of Nephrology, Department of Internal Medicine, Hsinchu Mackay Memorial Hospital, 690 Section 2, Guangfu Road, Hsinchu 300, Taiwan; 5 Division of Endocrinology, Department of Internal Medicine, Hsinchu Mackay Memorial Hospital, 690 Section 2, Guangfu Road, Hsinchu 300, Taiwan

**Keywords:** particulate matter (PM), PM_2.5_, animal model, in vitro study, disease progression

## Abstract

Air pollution is a very critical issue worldwide, particularly in developing countries. Particulate matter (PM) is a type of air pollution that comprises a heterogeneous mixture of different particle sizes and chemical compositions. There are various sources of fine PM (PM_2.5_), and the components may also have different effects on people. The pathogenesis of PM_2.5_ in several diseases remains to be clarified. There is a long history of epidemiological research on PM_2.5_ in several diseases. Numerous studies show that PM_2.5_ can induce a variety of chronic diseases, such as respiratory system damage, cardiovascular dysfunction, and diabetes mellitus. However, the epidemiological evidence associated with potential mechanisms in the progression of diseases need to be proved precisely through in vitro and in vivo investigations. Suggested mechanisms of PM_2.5_ that lead to adverse effects and chronic diseases include increasing oxidative stress, inflammatory responses, and genotoxicity. The aim of this review is to provide a brief overview of in vitro and in vivo experimental studies of PM_2.5_ in the progression of various diseases from the last decade. The summarized research results could provide clear information about the mechanisms and progression of PM_2.5_-induced disease.

## 1. Introduction

Particulate matter (PM) is a complex mixture of solid and liquid elements that are distributed in the air. Most fine PM (PM_2.5_) is from anthropogenic emissions and a few are from natural emissions, with the major sources of anthropogenic emissions being industry, coal combustion, and traffic pollution. The components of PM_2.5_ also exhibit variety in different seasons and cities. Accordingly, the composition, size, biological properties, and physical properties of particles are related to the region, season, and change over time [1,2]. Differences in pollution levels are generated from various pollution sources. The main components of PM are heavy metals, carbon sources, sulfate, ammonium, nitrate, and various ions. In ambient air, several gas pollutants are generated from the same sources as PM pollution, such as carbon monoxide, ozone, nitrogen oxides, and sulfur dioxide. Gas pollutants combined with PM might have adverse effects on human health. The adverse health effects of PM on the progression of diseases mainly depend on gender due to exposure differences, hormonal status, life stages, and other factors [3]. 

Fine PM, with a size of less than 2.5 μm, is known as PM_2.5_, which is mostly derived from combustion sources. The carbon core of PM_2.5_ is coated with hydrocarbons, metals ions, and secondary particles derived from nitrogen oxides and sulfur oxides. The large surface area of PM_2.5_ contributes to the combination of toxic compounds, including polycyclic aromatic hydrocarbons (PAHs), volatile organic compounds (VOCs), and transition metals [4,5]. PM_2.5_ can be inhaled into the gas exchange area of human lungs [6,7] where the ultrafine component is released to the systemic circulatory system, causing damage to cells and tissues [8,9,10]. 

## 2. Potential Mechanisms of PM_2.5_ in Disease Progression

**Figure 1:**
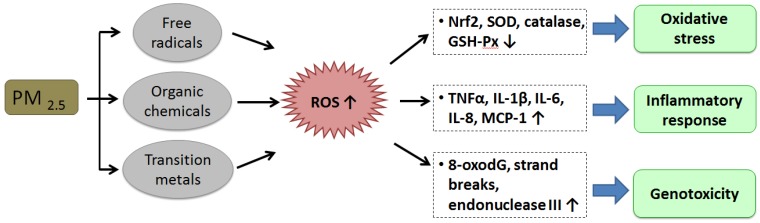
**The potential mechanisms of PM_2.5_ on disease progression.** The cellular toxicity components, mainly including free radicals, organic chemicals, and transition metals, of PM_2.5_ may induce or produce reactive oxygen species (ROS) that impair the cellular physiological/biochemical processes by the mechanisms of inducing oxidative stress, inflammation, genotoxicity, and others, altering the normal physiological functions and/or fates of target cells, resulting in damage of the tissues and organs.

PM_2.5_ is closely related to adverse health outcomes. The potential mechanisms of the impact of PM_2.5_ on the pathogenesis of diseases include oxidative stress, inflammatory responses, and genotoxicity (Figure 1). PM_2.5_-induced reactive oxygen species (ROS) have been regarded as a crucial mediator of PM_2.5_ toxicity [11,12,13]. Organic compounds and free radicals generated from combustion sources could be connected with PM_2.5_. Reactive electrophilic metabolites are the electrophilic messengers for ROS that activates ROS through redox cycling and metabolic activation. [14,15,16,17,18,19,20]. Transition metals, such as Mn, Vn, Cu, and Fe, coated to PM_2.5_ and the resulting Fenton reaction produces intracellular ROS. Oxidative stress can be induced by increasing ROS, which impairs the antioxidant system by decreasing the nuclear factor, erythroid-2-related factor 2 (Nrf2) [11], and reducing the antioxidant ability of the cells by reducing antioxidant enzymes, such as superoxide dismutase (SOD), glutathione peroxidase (GSH-Px), and catalase [21,22,23]. PM_2.5_ induces the activation of inflammatory cells, which can produce ROS due to the generation of oxidative stress in cells [24,25]. 

PM_2.5_-induced ROS enhances the gene and protein expression of pro-inflammatory factors, including tumor necrosis factor-α (TNF-α), interleukin-1β (IL-1β), interleukin-6 (IL-6), interleukin-8 (IL-8), and monocyte chemoattractant protein-1 (MCP-1). ROS also enhances inflammation in the pathogenesis of various diseases [26,27,28,29,30]. Inflammation has been shown to be involved in most, if not all, of the adverse health effects of PM_2.5_, and has demonstrated a central role in the impacts of PM_2.5_. In genotoxicity, augmented levels of 7-hydro-8-oxo-2′-deoxyguanosine (8-oxodG) are critical predictors of oxidative DNA damage. PM_2.5_-induced ROS is associated with several types of DNA damage through enhanced levels of strand breaks, 8-oxodG, and endonuclease III in animal and human cells [17,29,31,32].

In recent years, PM_2.5_ pollution has gradually drawn more attention, and several countries are investigating the relationships between PM_2.5_ and human health. A number of epidemiological review articles have shown that PM_2.5_ is associated with the pathogenesis of several chronic diseases, including respiratory system injuries [33,34], cardiovascular diseases [35,36], and diabetes mellitus [37]. Some review articles also summarize the potential mechanisms of PM_2.5_ in ambient air on adverse health outcomes [38,39,40,41]. However, the mechanisms underlying these effects have not been fully understood. Therefore, this review discusses experimental cell and animal studies that may help in understanding the suggested links between increased PM_2.5_ levels and the progression of disease in humans.

## 3. In Vitro Experimental Studies of PM_2.5_-Induced Cell Damage

In vitro cell investigations can be used as representatives of various cell types. Cells can readily be obtained from detailed mechanism research for embryos, tissues, and animal organs [42]. Previous research indicates that the effects of PM_2.5_ on human health are related to the size and components of the particles, which have a fundamental relationship with the progression of multiple diseases [43,44]. Cell experiments are a useful tool and rapidly provide information on each component of PM_2.5_ and its specific health effects. The main limitation of in vitro investigations is that cells have been removed from biological conditions. Most in vitro investigations are performed using a monolayer of one cell type that excludes interacting neighboring cells, despite intercellular signaling being essential for tissue and organ homeostasis [45]. It is especially important for freshly isolated cells to preserve the multidimensional structures and interactions with different cell types that normally form tissues [45,46,47]. In the complex environment of human organisms, using only one cell type is too different from the normal biological state. Therefore, the development of in vitro cell co-culture systems are a useful tool to examine true exposure to airborne PM.

Consequently, submerged cell culture is a common method to explore the adverse effects of PM_2.5_ in cell experiments. Air liquid interface (ALI) cell culture is another critical method and displays similar differentiation and growth states to those observed in vivo. ALI cell culture can restore the pseudostratified striation of the respiratory airway in vitro, so it is a useful culture method for investigating the harmful effects of PM_2.5_ on the respiratory epithelium [48]. We review the recent in vitro research that indicates the cellular effects of the major components of PM_2.5_. The findings demonstrate the relationships between specific cell damage and different PM_2.5_ components, as well as clarify the potential mechanisms (Table 1). The results could provide a foundation for future research on disease progression caused by PM_2.5_.

### 3.1. Pulmonary Diseases

The bronchial epithelium plays a role as a barrier and keeps the structure stable to protect the lungs in the respiratory system. The excretion of biological cytokines and other peripheral stimuli can lead to chronic and acute responses in this process. Human bronchial epithelial cells play an important role in biological effects under stress responses. Organic fractions (i.e., mainly PAHs) of PM_2.5_ induce gene and protein expression of pro-inflammatory cytokines in a time- and concentration-dependent manner. PAHs also cause micronucleus formation and DNA breakage, leading to the inhibition of antioxidant enzymes, including SOD, sodium selenite, mannitol, and catalase, in human bronchial epithelial cells (BEAS-2B) [49]. 

PM_2.5_ enhances autophagy mediated by the PI3K/Akt/mTOR pathway inhibition [50], inhibits P53 expression, and induces the hypermethylation of the P53 promoter through the ROS-Akt-DNMT3B pathway in BEAS-2B cells [51]. PM_2.5_ also induces the expression of the IL-8 gene by endocytosis and inducing oxidative stress in these cells [52]. Secondary organic aerosol-PM_2.5_ downregulates the expression and activation of the Nrf2-related transcription factor system in BEAS-2B cells [53]. Exposure of the ALI system to motorcycle exhaust increases oxidative stress and cytotoxicity. The use of a 0.20-μm PM filter dramatically reduces the particulate composition in PM and the concentration of total hydrocarbons. The filter displays protective effects by relieving the survival of exposed pulmonary epithelial cells and decreasing the ROS levels. Therefore, emission factors, such as different sizes of PM and total hydrocarbons from motorcycles, may play a role in motorcycle exhaust -related toxicity [54]. PM_2.5_-induced ROS also stimulates oxidative stress [55,56], apoptosis [57], and mitochondrial damage in 16-HBE cells [58]. 

PM_2.5_ could regulate the JAK/STAT signaling pathway and lead to oxidative damage in bronchial epithelial cells [55]. It also significantly induced oxidative damage, DNA strand breaks, and apoptosis by the p53-dependent pathway [57]. PM_2.5–0.3_ induces genetic instability and alterations of the cell cycle via TP53-RB gene signaling pathway activation in human alveolar macrophages and an L132 co-culture model [59]. Industrial PM_2.5_ extracts enhance inflammation and pulmonary epithelial cell injuries via the RhoA/ROCK-dependent NF-кB signaling pathway in a co-culture system of BEAS-2 and THP-1 cells [60]. 

PM_2.5_ enhances levels of arginase II via an EGF-related signaling pathway of oxidative stress and expression in human bronchial epithelial cells, which may be involved in the mechanism of adverse effects induced by PM exposure in asthma patients [61]. Tetra-OH-B[a]P and 8-OHdG levels are elevated in the DNA of primary human bronchial epithelial (HBE) cells from COPD patients compared to those in HBE from normal subjects. This indicates that COPD-DHBE cells were more sensitive to PM_2.5_ derived from air pollution [62]. Transition metals in PM increase the levels of IL-8 and HO-1, leading to ROS production in mucus-secreting ALI-cultured primary bronchial epithelial cells (PBECs) [63]. 

According to these studies, PM_2.5_ extracts impact various epithelial cells of the airway. Organic matter can affect the expression of crucial enzymes, and it influences the repair and synthesis of DNA. Organic extracts also play a key role in oxidative damage, the inflammatory response, and signaling pathways. Hydro-soluble fractions generate ROS, induce the secretion of inflammatory factors, and are related to genetic toxicity and the apoptotic response. Transition metals are related to genetic toxicity and inflammatory responses. 

A549 cells are lung cancer cells derived from human alveolar basal epithelial cells, with properties of both malignant tumor cells and alveolar type II cells. Therefore, A549 cells are commonly used in investigations of the development and treatment of lung cancer. Pavagadhi et al. (2013) studied the effects of PM_2.5_ samples from Singapore on A549 cells. 10 transition metals and 16 critical PAHs were analyzed in PM_2.5_ samples, and their biological effects included the induction of cell death and the reduction of cell viability [64]. 

Studies on organic and aqueous fractions of PM extracts showed that PM_2.5_ inhibited cell proliferation and that PM_10_ induced the release of lactate dehydrogenase (LDH) in A549 cells [65]. In the inflammatory response, extracts of PM_2.5_ upregulated biological factors that are associated with down-stream stimulation of kinase pathways and the caspase cascade. Upregulation of metal-redox-sensitive transcription factors, activator protein-1 (AP-1), and the transcription factor, κB (NF-κB), is correlated with a mechanism of cell death triggered via Fenton-active transition metal redox catalysis [66]. The autophagy might occur through the AMP-activated protein kinase signaling pathway [67]. 

PM_2.5_-induced ROS increases levels of intercellular adhesion molecule 1 (ICAM-1) through the IL-6/Akt/STAT3/NF-κB axis, which promotes monocyte adhesion to endothelial cells [68]. Recent studies suggest that PM_2.5_ from cooking oil fumes could lead to apoptosis in A549 cells and inflammation, which might occur through the activation of the MAPK/NF-кB/STAT1 signaling pathway [69]. PM_2.5_ enhances oxidative stress and cell cycle alteration in the G2/M phase, which is associated with increased p53 and p21 levels and decreased CDK1 mRNA expression in A549 cells [70]. PM_0.4_ and PM_1_ induce ROS generation and double-strand DNA breaks in a co-culture model of A549 and THP-1 cells, which may correlate with the exacerbation of respiratory diseases [71]. In conclusion, PM_2.5_ extracts–induced ROS play a crucial role in cellular autophagy and the apoptosis pathway. 

### 3.2. Cardiovascular Dysfunctions

Endothelial cells of vascular tissue form a tightly arranged layer on the surface of blood vessel linings. These cells regulate metabolism and the secretion functions of various physiological phenomena, including thrombosis prevention, blood volume, and blood pressure [72]. Metal ions of PM_2.5_ cause oxidative stress, resulting in PM_2.5_-mediated mitochondrial apoptosis via the NF-κB pathway in human umbilical vein endothelial cells (EA.hy926), which may enhance metal ions coated with endothelial cells [72]. PM_2.5_ stimulates oxidative stress and inflammation in endothelial cells; ERK1/2 is involved in the signaling pathway and leads to PM_2.5_-induced EA.hy926 endothelial cell injury [73]. 

Coal-fired PM_2.5_ from coal combustion has the potential to reduce cell viability, induce oxidative DNA damage, and induce global DNA methylation, and metal ions may be crucial factors that impact cellular toxicity in EA.hy926 cells [74]. PM_2.5_-induced ROS enhanced the levels of VCAM-1 and ICAM-1 via the ERK/Akt/NF-κB axis, which leads to monocyte adhesion to endothelial cells [75]. In a co-culture system of human umbilical vein endothelial cells and monocytic U937 cells, PM_2.5_ and PM_10_ enhanced monocytic adhesion via the expression of adhesion molecules, such as E-selectin, P-selectin, and ICAM-1, in the development of inflammatory responses, which may be used to evaluate the progression of atherosclerosis [76].

PM_2.5_ induced cell death and reduced cell viability via triggering of the MAPK signaling pathway and increasing intracellular ROS generation in rat H9c2 cells [77]. According to these results, metal ions of PM_2.5_ may be related to the induction of oxidative stress in endothelial cells. Organic soluble fractions of PM_2.5_ enhance the gene expression of antioxidant enzymes and oxidative stress. The dysfunction also affects the pathogenesis of various cardiovascular diseases, including hypertension, atherosclerosis, and diabetes. 

### 3.3. Immune Inflammatory Responses

Macrophages are a major target for the immune inflammatory responses caused by PM_2.5_. They play a central role in releasing cytokines after the inhalation of particles, and they participate in almost every immune inflammatory response. PM_2.5_ collected from six European cities increased the levels of chemokine (macrophage inflammatory protein 2), proinflammatory factors (TNF-α and IL-6), and NO production, and caused cytotoxicity in RAW264.7 [78]. PM_2.5_ can contain soil-derived Co, Cu from vehicular abrasives, and combustion-derived elements from fuel oil, such as V and Ni. These components significantly induced ROS levels in rat alveolar macrophage cells. The results suggest a dominant role of transition metals in generating ROS compared to organic carbon [79]. 

PM_2.5_ was collected and compared from two cities of New Zealand: Auckland and Christchurch. Their components were explored and correlated with the oxidative response in RAW264.7 macrophage cells. The concentrations of PAHs and hydro-soluble metal were higher in Christchurch PM_2.5_ than Auckland PM_2.5_. The organic fractions of Christchurch PM_2.5_ displayed Cyp1A1 activation and higher mutagenicity compared with Auckland OE-PM_2.5_. Hydro-soluble extracts of Auckland PM_2.5_ were more cytotoxic and led to greater activation of TNF-α release than those from Christchurch PM_2.5_ [80]. 

Studies report that PM_2.5_ induces autophagy of pulmonary macrophages by the ROS-related PI3K/Akt/mTOR pathway [81]. PM_2.5_ also could increase p38 and NF-κB levels in RAW 264.7 cells, leading to enhanced proinflammatory factors MCP-1 and TNF-α expression [82]. It also induces NO release and increases levels of LDH and intracellular ROS [83], which triggers the inflammatory response. Aqueous and organic fractions of PM_2.5_ induced the expression of the IL-1β through the TLR4/NF-κB axis, as well as the formation of the nucleotide-binding domain and leucine-rich repeat protein 3 inflammasome [84]. In a co-culture model of CD4+ T cell and dendritic cells, PM_2.5_ enhances the activation of dendritic cells and Th17-immune responses through the aryl hydrocarbon receptor-dependent pathway [85].

The above results show that the carbon core, hydro-soluble, hydro-insoluble, and lipid-soluble constituents of PM_2.5_ can result in injury to macrophages. Particularly, hydro-insoluble fractions, such as PAHs and VOCs, have a high correlation with mutation, cell toxicity, and the release of inflammatory cytokines. The metal elements of hydro-soluble constituents can lead to oxidative stress, and, transition metals particularly, show close correlations with the inflammatory response. Metal ions can also affect the antibacterial ability of macrophages. The interaction influences between each element may induce cell damage.

## 4. In Vivo Studies of PM_2.5_-Induced Damage by Animal Models

Animal experimental models are usually used to explore the possible health effects on humans due to environmental and occupational exposure to PM_2.5_. The high profile of the potential effects of occupational or environmental exposure to PM_2.5_ has led to numerous relevant studies on animals [86,87,88]. We attempted to summarize the in vivo experimental studies of PM_2.5_ on animal models regarding health effects, such as respiratory diseases, cardiovascular dysfunctions, diabetes mellitus, and allergic sensitization (Table 2).

The most common experimental species include mice, rats, and hamsters in exploring the effects of PM_2.5_. Due to the physical characteristics of their respiratory tracts, PM_2.5_ enters the alveolar cells. The major methods of PM_2.5_ exposure in animal models are intratracheal inhalation and intratracheal instillation. Both methods are in vivo methods that have strengths and weaknesses (Table 3). Inhalation exposure is a physiological method of exposure to PM_2.5_, and it requires an expensive exposure chamber, as well as technical experience. Intratracheal instillation involves applying the material directly to the trachea [89]. 

Intratracheal instillation is frequently used in mice, rats, and hamsters by inserting a needle into the mouth and throat. Compared to inhalation, intratracheal instillation is a more difficult operation that needs experienced animal trainers to inject the amount of liquid into the trachea and not the esophagus. It allows greater control over the concentrations and location of the material, and it is cheaper. The drawbacks of intratracheal instillation include its invasive and non-physiological nature, the fact that it bypasses the upper respiratory tract, and the confounding effects of the anesthesia and delivery vehicle [90]. 

### 4.1. Respiratory Diseases

The pulmonary system is a major target of PM_2.5_ effects. Many investigations demonstrate that PM_2.5_ can lead to an inflammatory response in the respiratory system [91,92], weakening of the pulmonary function [93,94], the incidence and deterioration of chronic obstructive pulmonary disease (COPD) and asthma [95,96,97], and making the lungs susceptible to infection [98,99]. Gender is also a vital factor in the relationship between PM_2.5_ and the pathogenesis of pulmonary disease. In a large lung health study, a higher prevalence of airway hyperresponsiveness was observed in female subjects among middle-aged smokers with mild COPD, which is due to the airway caliber in females being smaller than that in males [100]. Increased prenatal PM_2.5_ exposure at the mid-gestation stage was related to the progression of childhood asthma in boys [101]. Long-term SO_2_ exposure increases the risk of tuberculosis in men [102].

In human air-particulate exposure experiments, wood smoke can enhance the systemic inflammatory response and increase oxidative stress in the respiratory tract, especially in the lower airways of healthy people [103]. PAH-rich wood smoke particles induce DNA damage and cellular dysfunction, which deteriorate the airway’s inflammatory responses in humans [104]. Mouse-model studies show that desert-dust PM_2.5_ enhanced M2 macrophages and activated the Th2-mediated immune inflammatory response due to aggravation of pulmonary eosinophilia. In alveolar macrophages from mice, wood smoke PM_2.5_ induced v-rel reticuloendotheliosis viral oncogene homolog B activation and macrophage suppression, according to nuclear translocation [105]. These results indicate that low doses of PM_2.5_ may stimulate histological and functional changes in lung tissue, but do not impact oxidative stress and inflammation. 

Acute PM_2.5_ exposure enhances pulmonary oxidative stress and inflammatory responses and deteriorates pulmonary impedance in mice [106]. C57BL/6 mice that were exposed to PM_2.5_ demonstrated a significant inflammatory response via the elevation of MCP-1 and neutrophils in the lung tissue [107]. ACE2 knockdown will enhance inflammatory response, tissue remodeling and attenuate injury repair in PM_2.5_-induced acute lung injury via p-STAT3 and p-ERK1/2 signaling pathways [108]. Acute PM_2.5_ exposure triggered the Th2-mediated immune response, which leads to locally and systemically acute inflammations in mice, and TLR2 and TLR4 are associated with the progression, PM_2.5_ exposure reduced SOD and GSH-Px activities [109]. Endothelin (ET)-1 is the most potent endogenous bronchoconstrictor that plays a key role in vascular homeostasis. PM_2.5_ exposure upregulates endothelin A receptor and endothelin B receptor, which are associated with the JNK/p38 and MEK1/2/p38 pathways in rat bronchus cells [110]. PM_2.5_ can significantly enhance the changes of cyclin-dependent kinase 2 and p53 in the early phase, as well as cyclin B and cell cycle controller 2 in mid-term, and p21 in long-term exposure. Time is also a factor in the levels of PM_2.5_-related injury to lung tissue and the trachea [111]. 

In experimental models using rats, PM_2.5_ exposure significantly induced the expression of SOD, IL-6, IL-8, and neutrophil under cold stress. The results showed that oxidative stress and inflammatory responses were associated with the additive effect of cold stress and PM_2.5_ exposure on pulmonary injury [112]. PM_2.5_ can induce the inflammation response and reduce the phagocytic activity of alveolar macrophages, resulting in lung injuries in female Wistar rats [113]. 

PM_2.5_-induced pathological injury is related to ROS production, mitochondrial fusion–fission dysfunction, mitochondrial lipid peroxidation, and abnormal cellular homeostasis [114]. Moreover, maternal PM_2.5_ exposure may also upregulate the epithelial-mesenchymal transition ion through the TGF-β/Smad3 signaling pathway in postnatal pulmonary dysfunction [115]. 

### 4.2. Cardiovascular Dysfunctions

Several investigations have shown that PM_2.5_ exposure is not only related to cardiovascular function, but also the morbidity and mortality in cardiovascular disease [116,117,118]. Age, gender, and hormonal status are impact mediators in the modification of vascular toxicity by phenanthraquinone extracted diesel exhaust [119]. A meta-analysis in China reported that elevated air pollution is correlated with increased cardiovascular mortality, and lower temperature, age > 65 years, and being female were related to higher risks of cardiovascular mortality [120]. 

In human exposure studies, wood-smoke PM_2.5_ particles in smoky indoor environments seem to affect coagulation, inflammation, thrombosis, and lipid peroxidation. These factors may be associated with the mechanisms of PM in morbidity and mortality in cardiovascular disease [121,122]. In mouse studies, PM_2.5_ induced inflammatory responses in the myocardium by increasing the T helper 17-based perforin response, viral replication, and the ratio of abnormal matrix metalloproteinases 2 (MMP-2) to tissue inhibitor of metalloproteinases-1 (TIMP-1). PM_2.5_ also aggravated virus-related myocarditis, possibly via immune response depression [123] and T regulatory cell responses [124]. In a European study, PM_2.5_ exposure increased HO-1, endothelin-1 (ET-1), Cyp1A1, Cyp1B1 myeloperoxidase, and Hsp70 levels of the lung and heart in BALB/c mice [125]. 

ApoE^-/-^ mice are a useful model to investigate atherosclerotic disease. PM_2.5_ from motor vehicle traffic contains numerous components, such as residual oil, secondary sulfate, and resuspended soil, which depend on the time of day. The PM_2.5_ composition and different time periods are related to the alteration of heart rate and HR variability in ApoE^-/-^ mice. [126]. PM_2.5_ exposure could significantly increase malondialdehyde, reduce heart rate variability [127], and upregulate visfatin [128], which are conducive to oxidative stress of the heart and atherosclerosis. PM_2.5_ also induced CD36-dependent 7-ketocholesterol accumulation in macrophages in the progression of atherosclerosis [129]. The IκB kinase (IKK)/NFκB pathway also played a critical role in mediating the PM_2.5_-related cardiovascular impairment in a mouse model of type 2 diabetes mellitus [130]. 

PM_2.5_ induced systemic inflammation in Wistar rats [131]. PM_2.5_ exposure also depressed the cardiovascular system with diet-induced metabolic syndrome [132] and induced oxidative stress, inflammation, and calcium homeostasis disorder, resulting in mitochondrial damage in Sprague-Dawley rats [133]. In rat coronary arteries, PM_2.5_ induces the upregulation of endothelin B and endothelin A receptors via the MEK/ERK1/2 pathway [134]. In SD male rats, PM_2.5_ might exaggerate the neurobehavioral alterations associated with astrocyte activation and inflammatory reactions in ischemic stroke [135]. 

### 4.3. Diabetes Mellitus

Early evidence shows that PM_2.5_ exposure exacerbates the decline of renal function, which is related to time. Recently, chronic PM_2.5_ exposure has also been found to promote the progression of diabetes mellitus, including visceral adipose inflammatory responses, hepatic endoplasmic reticulum stress, brown adipose mitochondrial variations, and insulin resistance [136,137]. Furthermore, even low levels of PM_2.5_ exposure could increase the risk of mortality in diabetes mellitus [137]. PM_2.5_ is associated with a higher susceptibility to diabetes mellitus in women (adjusted HR, 1.17; 95%CI, 1.03–1.32), but not in men (adjusted HR, 1.03; 95%CI, 0.91–1.16) [138]. Furthermore, the prevalence of diabetes mellitus increases with an increasing of PM_2.5_ concentrations, with a rise of 4.0% (1.5–6.4%) for women and 3.5% (1.3–5.6%) for men per unit of elevated PM_2.5_ [139].

Epidemiological and experimental investigations reveal a relationship between insulin resistance and PM_2.5_ exposure, and the activation of innate immune responses may play a crucial role in the pathological progression of these effects. In type 2 diabetes patients, exposure to ultrafine elemental carbon particles enhances vascular endothelium and blood platelet activation, which shows that airborne particles could increase the risk for adverse cardiovascular effects in diabetes patients [140]. In C57BL/6 mice, long-term PM_2.5_ exposure induced impairment of glucose tolerance, insulin resistance, inflammation, and mitochondrial changes in the progression of type 2 diabetes. Chronic PM_2.5_ exposure also enhanced gene expression, mitochondrial alterations, and oxidative stress in brown and white adipose tissues [141]. PM_2.5_ induces NF-κB-related inflammasome activation and vascular insulin resistance leads to peripheral blood and bone marrow endothelial progenitor cells level recovery [142]. Chronic PM_2.5_ exposure induced macrophage infiltration, unfolded protein response activation, and enhanced gene expression of adipocyte differentiation, lipogenesis, and lipid droplet generation in the white adipose tissue of C57BL/6 mice. [143]. CC-chemokine receptor 2 (CCR2) induced systemic cellular inflammatory responses play a critical role in diet-induced insulin resistance. In HFD-fed CCR2^-/-^ male mice, PM_2.5_ enhanced insulin resistance through the regulation of hepatic lipid metabolism, inflammatory responses in visceral adipose tissue, and glucose utilization in skeletal muscle through both CCR2-dependent and independent pathways [144]. Exposure to PM_2.5_ markedly promoted [eHsp72]/[iHsp70] and the cell stress response, leading to increased metabolic dysfunction and risk for type 2 diabetes mellitus in HFD-fed mice [145]. 

In Sprague-Dawley rats, PM_2.5_ exposure significantly elevated the levels of glycated hemoglobin A1c, IL-6, and fibrinogen, which led to the deterioration of tubular injury, glomerulosclerosis, aortic medial thickness, and focal myocarditis in the kidney and heart [146]. PM_2.5_ exposure affected the angiotensin/bradykinin systems, immune system, and antioxidant imbalance in early kidney damage [147]. In PM_2.5_-induced hypertension, long-term PM_2.5_ exposure increases blood pressure by inhibiting D1 receptor-related sodium secretion through the regulation of G protein-coupled receptor kinase 4 in Sprague-Dawley Rats. [148]. In a rat model of gestational diabetes mellitus, PM_2.5_ exposure significantly reduced the levels of GSH-Px and induced malondialdehyde, resulting in an oxidative response and inflammation in the pancreas. Furthermore, pancreatic GLUT2 levels declined after PM_2.5_ exposure [149].

### 4.4. Allergic Sensitization

In mice and humans, studies demonstrate that numerous types of particles induce allergic inflammation [150]. Both the organic and inorganic constituents that coat particles and the particle cores have been shown to enhance allergic sensitization [151,152,153]. The components and solubility of particles have also been shown to play an important role in allergic sensitization [154]. In NC/Nga mice, which have a high susceptibility to mite allergens, PM_2.5_ can enhance allergic airway inflammation through inflammasome activation and the synergistic action of insoluble and soluble fractions of PM_2.5_ [155]. 

Acute exposure to PM_2.5_ could synergize with allergens to exacerbate the progression of asthma via activation of the Th2-related immune response in ovalbumin-sensitized mice [156]. Combined exposure to PM_2.5_ and formaldehyde could significantly exacerbate allergic asthma, which is associated with induced oxidative stress via the transient receptor potential vanilloid 1 signaling pathway [157], and also through thymic stromal lymphopoietin activation in mice [158]. In a recent report, PM_2.5_ and allergens from dust mites enhanced the hyper-responsiveness of the airway through the activation of T-helper cell type 17 (TH17) activation [159]. In a guinea pig model, acute PM_2.5_ exposure with aluminum hydroxide in sensitized animals enhanced the specific-hyperresponsiveness and eosinophilic and neutrophilic airway inflammation in allergic asthma [160].

## 5. Conclusions

PM_2.5_ air pollution is a major cause of morbidity and mortality around the world. Air pollution control and pathogenesis exploration of PM_2.5_ are extremely important issues. Oxidative stress, inflammation, and genotoxicity are the main potential mechanisms in PM_2.5_-induced disease progression. The research findings of in vitro cell and in vivo animal investigations have provided vital insights into the mechanisms of PM_2.5_ exposure in disease progression. Better understandings of the disease mechanisms associated with PM_2.5_ will allow the development of new strategies to help people who are at risk and to decrease the harmful effects of PM_2.5_ on the pathogenesis of various diseases. To better address the knowledge gaps; the focus of exploration should be on the molecular mechanisms by which PM_2.5_ and its components affect public health.

## Figures and Tables

**Table 1:** Summary of in vitro studies for the effects of PM_2.5_ treatment on cell responses and damages.

Diseases	Cell Line	Dosage	Cell Cultured Method	Study Findings and/or Conclusion	Reference
	BEAS-2B	50 mg/mL	submerged	Organic fraction of PM_2.5_ causes micronucleus formation and DNA breakage leads to inhibition of antioxidant enzymes, which increases the oxidative stress.	[49]
		25, 50, and 100 µg/mL	submerged	PM_2.5_ enhances autophagy via PI3K/Akt/mTOR pathway inhibition.	[50]
		1.5, 3, and 6 μg/cm^2^	submerged	PM_2.5_ inhibits the P53 levels through ROS-Akt-DNMT3B pathway-related p53 promoter hypermethylation.	[51]
		2.5, 5, and 10 μg/cm^2^	submerged	PM_2.5_ induces expression of the IL-8 gene by endocytosis and oxidative stress induction.	[52]
		0.1 mg/mL	air-liquid interface	Secondary organic aerosol-PM_2.5_ downregulates the expression and activation of Nrf2-related transcription factor system.	[53]
		289.4 μg/m^3^	air-liquid interface	Motorcycle exhaust-PM exposure reduces cell relative viabilities and induces ROS generation	[54]
	16HBE cells	50 and 100 μg/mL	submerged	PM_2.5_ can regulate JAK/STAT signaling pathway leading to oxidative damage of cells	[55]
		50 µM/cm^2^	submerged	PM_2.5_ induces the Der p1 antigen-related innate immune response via the increasing of IL-25, IL-33, and TSLP levels.	[56]
		16, 32, 64, and 128 mg/mL	submerged	PM_2.5_ induces oxidative damage, DNA strand breaks, and apoptosis occurs by the p53-dependent pathway.	[57]
		100 mg/mL	submerged	PM_2.5_ elevates ROS generation, and inhibits mitochondrial genes expressions, resulting in mitochondrial damage and apoptosis.	[58]
	Human AM/L132	18.84, 37.68, 56.52, 75.36, and 150.72 μg/mL	submerged	PM_2.5-0.3_ induces genetic instability and alterations of cell cycle via TP53-RB gene signaling pathway activation in the human alveolar macrophage and L132 co-culture model	[59]
	BEAS-2/THP-1	10 and 100 μg/mL	submerged	Industrial PM_2.5_ extracts enhance inflammation and pulmonary epithelial cells injuries via the RhoA/ROCK-dependent NF-кB signaling pathway.	[60]
	HBEC	100 μg/mL	air-liquid interface	PM_2.5_ enhances arginase II levels via the EGF-related signaling pathway of oxidative stress and expression in human bronchial epithelial cells.	[61]
		1, 10, and 100 μg/cm^2^	air-liquid interface	PM_2.5_ elevates the tetra-OH-B[a]P and 8-OHdG levels in the DNA of primary human bronchial epithelial (HBE) cells from COPD patients compared to those in HBE from normal subjects.	[62]
	PBEC	1.1, 2.2, 5.6, and 11.1 μg/cm^2^	air-liquid interface	Transition metals of PM increase the levels of IL-8 and HO-1, leading to ROS production in mucus-secreting ALI-cultured primary bronchial epithelial cells.	[63]
	A549	＿	submerged	10 transition metals and 16 critical PAHs were analyzed in PM_2.5_ samples, and their biological effects included the induction of cell death and the reduction of cell viability	[64]
		50, 100, and 200 μg/mL	submerged	Organic and aqueous fraction of PM extracts inhibit cell proliferation by PM_2.5_ and induces LDH release by PM_10_.	[65]
		25, 50, 100, 200, 300, and 600 μg/mL	submerged	PM_2.5_ upregulates the metal-redox-sensitive transcription factors, NF-κβ and AP-1 in apoptosis.	[66]
		100 μg/mL	submerged	PM_2.5_-induces autophagy via the AMP-activated protein kinase signaling pathway.	[67]
		50 and 100 μg/mL	submerged	PM_2.5_-induces ROS increases ICAM-1 levels through the IL-6/Akt/STAT3/NF-κB axis.	[68]
		75 μg/mL	submerged	Cooking oil fumes-PM_2.5_ can lead A549 cells to apoptosis and inflammation that might be through the activation of the MAPK/NF-кB/STAT1 signaling pathway.	[69]
		12.5, 25, 50, 100, and 200 μg/mL	submerged	PM_2.5_ enhances oxidative stress and cell cycle alteration in theG2/M phase that is associated with increased p53 and p21 levels and decreased CDK1 mRNA expression in A549 cells.	[70]
	A549/THP-1	10 μg/cm^2^	submerged	PM_0.4_ and PM_1_ induce ROS generation and double strand DNA breaks in A549 and THP-1 cells co-culture model that may correlate with the exacerbation of respiratory diseases.	[71]
Cardiovascular dysfunctions	EA.HY926	0.01, 0.1 and 1 mg/cm^2^	submerged	Metal ions of PM_2.5_ cause oxidative stress, resulting in PM_2.5_-mediated mitochondrial apoptosis via the NF-κB pathway.	[72]
		0,20, 200, 400 mg/L	submerged	PM_2.5_ stimulates oxidative stress and inflammation in endothelial cells; ERK1/2 is involved in the signaling pathway.	[73]
		10, 25, and 50 μg/mL	submerged	Coal-fired PM_2.5_ has the potency to reduce cell viability, induce oxidative DNA damage, and global DNA methylation.	[74]
		25, 50, 100, and 200 μg/mL	submerged	PM_2.5_-induced ROS enhances VCAM-1 and ICAM-1 levels via the ERK/Akt/NF-κB axis that leads to monocyte adhesion to endothelial cells.	[75]
	HUVEC/U937	5, 10, 20, and 40 μg/cm^2^	submerged	PM_2.5_ and PM_10_ enhance monocytic adhesion via the expression of adhesion molecules, such as E-selectin, P-selectin, and ICAM-1, in the development of inflammatory responses, which may evaluate the progression of atherosclerosis	[76]
	H9c2 cells	100, 200, 400 and 800 μg/mL	submerged	PM_2.5_ induces cell death and reduces cell viability via triggering of the MAPK signaling pathway and increasing intracellular ROS generation.	[77]
Immune inflammatory responses	RAW 264.7	＿	submerged	PM_2.5_ collected from six European cities increases the levels of chemokine (macrophage inflammatory protein 2), proinflammatory factors (TNF-α and IL-6), and NO production, and caused cytotoxicity.	[78]
		＿	submerged	Transition metals (Co, Cu, V and Ni) of PM_2.5_ significantly induces ROS levels.	[79]
		＿	submerged	Organic fractions of PM_2.5_ display Cyp1A1 activation and higher mutagenicity and led to greater activation of TNF-α release.	[80]
		25, 50, and 100 μg /mL	submerged	PM_2.5_ induces autophagy of pulmonary macrophages via the oxidative stress-mediated PI3K/AKT/mTOR pathway.	[81]
		30 μg /mL	submerged	PM_2.5_ induces NF-κB and p38 levels, leading to enhanced proinflammatory factors, and MCP-1 and TNF-αexpression, which triggers inflammatory responses.	[82]
		50, 100, and 200 mg/mL	submerged	PM_2.5_ induces NO release and increases levels of LDH and intracellular ROS.	[83]
		100 μg/mL	submerged	Aqueous and organic fractions of PM_2.5_ induce expression of IL-1β through the TLR4/NF-κB axis, conducive to nucleotide-binding domain and leucine-rich repeat protein 3 inflammasome formation.	[84]
	CD4^+^ T/ DC	50 µg/mL	submerged	PM_2.5_ enhances the activation of dendritic cells and Th17-immune responses through the aryl hydrocarbon receptor-dependent pathway.	[85]

**Table 2:** Summary of in vivo studies on health effects of PM_2.5_.

Health Effects	Animal Models	PM_2.5_ Dosage	Methods of PM_2.5_ Treatment	Study Findings and/or Conclusion	Reference
Respiratory diseases	C57BL/6 mice	5 and 15 μg/mouse	Instillation	Low doses of PM_2.5_ may simulate histological and functional changes in lung tissue, but do not impact oxidative stress and inflammations. Acute PM_2.5_ exposure enhances pulmonary oxidative stress, inflammatory responses, and deteriorated pulmonary impedance.	[106]
		6 h/day, 5 days/week for 5, 14, and 21 days	Inhalation	PM_2.5_ induces MCP-1 and neutrophils levels in pulmonary inflammation.	[107]
		6.25 mg/kg/day, once a day for 3 days	Instillation	ACE2 deficiency enhances inflammatory responses and tissue remodeling through p-STAT3 and p-ERK1/2 signaling pathways in PM_2.5_-induced acute lung injury.	[108]
		2.5, 5, and 10 mg/kg/mouse	Instillation	Acute PM2_.5_ exposure triggered the Th2-mediated immune response and leads to locally and systemically acute inflammations in mice.	[109]
	Wistar rats	0.3, 1.0, and 3.0 μg /mL	Instillation	PM_2.5_ upregulates endothelin A receptors and endothelin B receptors, and are associated with JNK and p38 pathways and MEK1/2 and p38 pathways in rat bronchi.	[110]
		0.4 mg/mL/rat	Inhalation	PM_2.5_ can significantly enhance changes of Cyclin-dependent kinase 2 and p53 in the early phase, cyclin B and Cell Cycle Controller 2 in mid-term, and p21 in long-term exposure.	[111]
		8 mg/rat	Instillation	PM_2.5_ exposure significantly induces the expression level of SOD, IL-6 and IL-8, and neutrophil under cold stress.	[112]
		0.3, 0.75, 2, and 5 mg/rat	Instillation	PM_2.5_ can induce the inflammation response and reduce the phagocytic activity of alveolar macrophages, resulting in lung injuries in female rats.	[113]
		0.375, 1.5, 6.0, and 24.0 mg/kg (body weight)	Inhalation	PM_2.5_-induced pathological injury is related to ROS production, mitochondrial fusion–fission dysfunction, mitochondrial lipid peroxidation, and abnormal cellular homeostasis	[114]
		0.1, 0.5, 2.5, and 7.5 mg/kg	Intraperitoneal instillation	Maternal PM_2.5_ may upregulate the epithelial-mesenchymal transition ion through the TGF-β/Smad3 signaling pathway in postnatal pulmonary dysfunction.	[115]
Cardiovascular dysfunction	Balb/c mice	10 mg/kg	Instillation	PM_2.5_ increases T helper 17-mediated viral replication, perforin response, and imbalance of MMP-2/TIMP-1 in virus-induced myocarditis.	[123]
		10 mg/kg	Instillation	PM_2.5_ induces T regulatory cells (Treg) responses in virus-induced myocarditis.	[124]
		0.3 mg/mouse	Instillation	PM_2.5_ exposure induces HO-1, ET-1, Cyp1A1, Cyp1B1 myeloperoxidase, and Hsp70 levels of lung and heart.	[125]
	Apoe(-/-) mice	6 h/day, 5 days/week for 6 months	Inhalation	Ni and P of PM_2.5_ may correlate with heart rate and heart rate variability. Long-range transported PM_2.5_ may positively associate with heart rate and negatively with heart rate variability.	[126]
		3, 10, and 30 mg/kg	Instillation	PM_2.5_ can significantly enhance malondialdehyde and reduce heart rate variability in atherosclerosis.	[127]
		24 h/day, 7 days/week, for 2 months	Inhalation	PM_2.5_ can upregulate the visfatin to activate the inflammation, oxidative stress and accelerate the atherosclerosis.	[128]
		6 h/day, 5 days/week for 3 months	Inhalation	PM_2.5_ induces CD36-dependent 7-ketocholesterol accumulation in macrophages on the progression of atherosclerosis.	[129]
	Kkay mice	6 h/day, 5 day/week for 8 weeks	Inhalation	IKK/NFκB pathway also plays a critical role in mediating PM_2.5_-related cardiovascular impairment in a type 2 diabetes mellitus mice model.	[130]
	Wistar rats	0.2, 0.8, and 3.2 mg/rat	Instillation	PM_2.5_ alone exposure induces inflammation, endothelial function, and ANS injuries, and ozone potentiated these effects induced by PM_2.5_.	[131]
	SD rats	10 h/day for 4 or 5 consecutive days	Inhalation	PM_2.5_ exposure depresses cardiovascular system with diet-induced metabolic syndrome.	[132]
		0.375, 1.5, 6, and 24 mg/kg	Instillation	PM_2.5_ induces oxidative stress, inflammation, and calcium homeostasis disorder, resulting in mitochondrial damage.	[133]
		0, 0.3, 1, and 3 mg/mL for 24 h	Instillation	PM_2.5_ induces the endothelin B and endothelin A receptor upregulation via the MEK/ERK1/2 pathway in rat coronary arteries.	[134]
		10 mg/mL/day for 7 days	Nasal Inoculation	PM_2.5_ might exaggerate neurobehavioral alterations that are associated with astrocytes activation and inflammatory reactions in ischemic stroke of SD male rats.	[135]
Diabetes mellitus	C57BL/6 mice	6 h/day, 5 days/week for 10 months	Inhalation	Chronic PM_2.5_ exposure enhances gene expression, mitochondrial alterations, and oxidative stress in brown and white adipose tissues.	[141]
		6 h/day, for 9 or 30 consecutive days	Inhalation	PM_2.5_ induces NF-κB-related inflammasome activation and vascular insulin resistance leads to peripheral blood and bone marrow endothelial progenitor cells level recovery.	[142]
		6 h/day, 5 days/week for 10 months	Inhalation	Chronic PM_2.5_ exposure induces macrophage infiltration and Unfolded Protein Response in white adipose tissue.	[143]
		6 h/day, 5 days/week for 17 weeks	Inhalation	PM_2.5_ enhances insulin resistance through regulation of hepatic lipid metabolism, visceral adipose tissue inflammatory responses, and glucose utilization in skeletal muscle through both CCR2-dependent and -independent pathways in HFD-fed mice.	[144]
	B6.129SF2/J mice	5 μg/day/mouse for 12 weeks	Instillation	PM_2.5_ promotes [eHsp72]/[iHsp70] and the cell stress response, leading to an increased risk of metabolic dysfunction and type 2 diabetes mellitus in HFD-fed mice	[145]
	SD rats	24 h/day, 7 days/week, for 16 weeks.	Inhalation	PM_2.5_ exposure significantly elevates the levels of glycated hemoglobin A1c, IL-6, and fibrinogen, which lead to the deterioration of tubular injury, glomerulosclerosis, aortic medial thickness, and focal myocarditis in the kidney and heart.	[146]
		5 h/day for 3 days	Inhalation	PM_2.5_ exposure increases the angiotensin/bradykinin systems, immune, and antioxidant imbalance in early kidney damage.	[147]
		3 and 30 μg/mouse	Instillation	Long-Term PM_2.5_ increases blood pressure by inhibition of the D1 receptor through regulation of the G protein-coupled receptor, kinase 4	[148]
		15 mg/kg, cumulative dose is 30 mg/kg	Instillation	PM_2.5_ reduces levels of GSH-Px and induced malondialdehyde, resulting in an oxidative response and inflammation in the pancreas, and pancreatic GLUT2 levels declined.	[149]
Allergic sensitization	NC/Nga mice	Supernatant fraction: 50 μg; precipitate fraction: 200 μg	Nasal Inoculation	PM_2.5_ can enhance airway hyperresponsivness in mice through an inflammasome activation and synergistic action of insoluble and soluble fractions of PM_2.5_.	[155]
	Balb/c mice	1, 10, and 100 μg/mouse	Instillation	PM_2.5_ can synergize with allergens to exacerbate the progression of asthma via activation of the Th2-related immune response.	[156]
		100 μg/mouse	Intraperitoneal injection	PM_2.5_ and formaldehyde co-exposure can induce oxidative stress to significantly exacerbate allergic asthma via the transient receptor potential vanilloid 1 pathway.	[157]
		10, 31.6, or 100 µg/mouse	Instillation	PM_2.5_ exacerbates allergic airway inflammation via thymic stromal lymphopoietin activation.	[158]
		33.3 µg/mouse	Instillation	PM_2.5_ and allergens from dust mites enhance the hyper-responsiveness of the airway through the activation of T-helper cell type 17 activation.	[159]
	Guinea pig	1.1 ± 0.2 kg/chamber	Inhalation	Acute PM_2.5_ exposure with aluminum hydroxide in sensitized animals enhances the specific-hyperresponsiveness and eosinophilic and neutrophilic airway inflammation in allergic asthma.	[160]

**Table 3:** Strengths and weaknesses of intratracheal inhalation and intratracheal instillation on PM_2.5_ exposure to experimental rodents.

Methods	Intratracheal Instillation	Intratracheal Inhalation
Operative difficulty	High	Low
Equipment cost	Low	High
Dosage	Instillated dosage (mg/kg of body weight or mg/animal).	Real deposition in the respiratory system of animal model. Defined by the PM_2.5_ concentration (mg/m^3^)
Deposition	Uneven distribution in the lung lobes	Evenly distributed in the lung lobes
Effects for animal	More severe Only affects lower respiratory tract	Less severe Affects whole respiratory tract

## Abbreviations

8-oxod	G 7-hydro-8-oxo-2′-deoxyguanosine
COPD	chronic obstructive pulmonary disease
Cyp	cytochrome P450
GSH-Px	glutathione peroxidase
Hsp72	heat shock protein 72
HO-1	heme oxygenase-1
ICAM-1	intercellular Adhesion Molecule 1
IL-1β	interleukin-1β
IL-6	interleukin-6
IL-8	interleukin-8
LDH	lactate dehydrogenase
MCP-1	monocyte chemoattractant protein-1
MMP-2	matrix metalloproteinases 2
NF-κB	transcription factor kappab
NO	nitric oxide
Nrf2	nuclear factor erythroid-2-related factor 2
OE-DEP	organic extract of diesel exhaust particles
PM	particulates matter
PAHs	polycyclic aromatic hydrocarbons
ROS	reactive oxygen species
SOD	superoxide dismutase
TIMP-1	tissue inhibitors of metalloproteinases-1
TNF-α	tumor necrosis factor-α
VOCs	volatile organic compounds
